# Measuring human cerebral blood flow and brain function with fiber-based speckle contrast optical spectroscopy system

**DOI:** 10.1038/s42003-023-05211-4

**Published:** 2023-08-14

**Authors:** Byungchan Kim, Sharvari Zilpelwar, Edbert J. Sie, Francesco Marsili, Bernhard Zimmermann, David A. Boas, Xiaojun Cheng

**Affiliations:** 1 Neurophotonics Center, Department of Biomedical Engineering, Boston University, Boston, MA USA; 2 Reality Labs Research, Meta Platforms Inc, Menlo Park, CA USA

**Keywords:** Blood flow, Translational research

## Abstract

Cerebral blood flow (CBF) is crucial for brain health. Speckle contrast optical spectroscopy (SCOS) is a technique that has been recently developed to measure CBF, but the use of SCOS to measure human brain function at large source-detector separations with comparable or greater sensitivity to cerebral rather than extracerebral blood flow has not been demonstrated. We describe a fiber-based SCOS system capable of measuring human brain activation induced CBF changes at 33 mm source detector separations using CMOS detectors. The system implements a pulsing strategy to improve the photon flux and uses a data processing pipeline to improve measurement accuracy. We show that SCOS outperforms the current leading optical modality for measuring CBF, i.e. diffuse correlation spectroscopy (DCS), achieving more than 10x SNR improvement at a similar financial cost. Fiber-based SCOS provides an alternative approach to functional neuroimaging for cognitive neuroscience and health science applications.

## Abstract (web summary)

Fiber-based speckle contrast optical spectroscopy measures human brain activation via cerebral blood flow changes, outperforming current methods.

## Introduction

Cerebral blood flow (CBF) is an important indicator of brain health as it regulates oxygen delivery to the brain and removes metabolic waste such as carbon dioxide. Alterations in CBF correlate with serious clinical conditions such as ischemic stroke^1,2^, traumatic brain injury^3^, and Alzheimer’s disease^4,5^. CBF also provides information about brain function^6–9^ as neural activation induces hemodynamic changes via neurovascular coupling^10^. Thus, monitoring CBF is important for cognitive neuroscience studies as well as clinical applications. Diffuse correlation spectroscopy (DCS) is an optical technique that measures human CBF from coherent light re-emitted from the tissue^11–15^. The blood flow index (BFi), a metric linearly correlated with underlying blood flow, is calculated from the decorrelation time of the autocorrelation function of the speckle intensity time course. It offers a convenient way to non-invasively and continuously monitor CBF at the bedside that cannot be accomplished with other techniques such as positron emission tomography and arterial spin labeling magnetic resonance imaging. However, traditional DCS systems suffer from a relatively low signal-to-noise ratio (SNR), and the single-photon avalanche diode (SPAD) detectors used in these systems are generally expensive, making it challenging for high density geometries covering large brain regions, or to average over multiple speckles/channels to improve SNR. Recently, several groups have attempted to improve DCS SNR by either imaging multiple speckles onto a SPAD array or improving the photon flux detection per speckle. For example, a recently published work on multi-speckle DCS with 1,024 parallel detection channels^9,16^ showed promising improvements in SNR, and demonstrated human forehead CBF variations at a short source detector separation (SDS) of *ρ* = 15 mm. But at *ρ* = 15 mm, the sensitivity to the brain is low and not feasible for measuring brain function^17^. In another example, implementing interferometry has been shown to improve DCS SNR by achieving shot noise performance^18,19^, but at the expense of increased complexity of the system which is not preferred for the future development of wearable devices. Finally, using a longer wavelength of 1064 nm as the input light source has also been shown to raise DCS SNR by increasing photon flux, thanks to the higher maximum permissible exposure (MPE) and lower energy per photon than that of shorter wavelengths, but this method requires even more costly superconducting nanowire single-photon detectors^20^.

Another category of optical techniques to measure CBF is laser speckle contrast imaging (LSCI)^21–24^. Instead of analysing the temporal statistics i.e. auto-correlation function of the speckle intensities as done in DCS, LSCI exploits the spatial statistics by calculating the spatial contrast of the speckle intensity patterns measured within a certain camera exposure time. The speckle intensity patterns are obtained using relatively low-cost complementary metal–oxide–semiconductor (CMOS) cameras that can capture millions of speckles with millions of pixels to improve SNR as opposed to SPADs used in traditional DCS that utilize a few speckles. However, traditional LSCI has primarily been used to obtain two-dimensional images of superficial CBF with wide-field illumination, mostly for small animals such as mice with cranial windows or thinned skulls. Recently, a technique derived from LSCI named speckle contrast optical spectroscopy (SCOS) and its tomographic expansion speckle contrast optical tomography (SCOT) have been demonstrated for free-space imaging with larger SDSs, enabling non-invasive measurements of blood flow in deeper regions in phantoms, human arms and forehead, and small animal brains^25–30^. However, generalizing free space techniques to human brain function measurements over a large area is challenging due to the presence of hair, susceptibility to motion due to limited range of focus, and the limited field of view of the camera. Fiber-based systems have been proposed and utilized to conduct measurements of cardiac pulse waveforms^31–33^, but the measurement of human brain function at a large source-detector separation (>30 mm) with comparable or greater sensitivity to cerebral rather than extracerebral blood flow has not yet been achieved. In addition, various noise sources will induce bias in the measured spatial contrast in SCOS, which presents challenges for quantifying blood flow changes at the low photon flux regime typically encountered for human CBF measurements^34^. Pioneering work on the correction of shot and dark noise has been modeled and utilized experimentally^28,34^, but there are more noise sources such as inhomogeneity in the illumination and quantization noise which need to be corrected for. Moreover, the noise correction scheme for human brain measurements has not been validated experimentally.

Here, we developed a fiber-based SCOS system capable of measuring human CBF and brain function non-invasively. We created and experimentally validated a data analysis pipeline to remove the bias in the contrast induced by noise. We have utilized a laser source pulsing strategy to improve the photon flux^35^ within the camera exposure time while keeping the average as well as the peak power within the safety limit. We verified experimentally that the current design of our fiber-based SCOS system using a scientific CMOS (sCMOS) camera outperforms traditional DCS systems by an order of magnitude in SNR at a comparable cost. We performed human brain function measurements for the first time using SCOS during a mental subtraction task at *ρ* = 33 mm, providing sufficient sensitivity to CBF variations. This work opens an avenue for future development of high-channel count and high-density optical devices (for tomography measurements) that can continuously monitor human CBF and brain function with unprecedented signal quality at large SDSs. The cost can be further reduced in the future by using lower cost CMOS cameras compared to the sCMOS camera used in this study. Continuous monitoring of CBF with high signal quality offers more opportunities for biology, cognitive neurosciences and clinical applications.

## Results

**Fig. 1:**
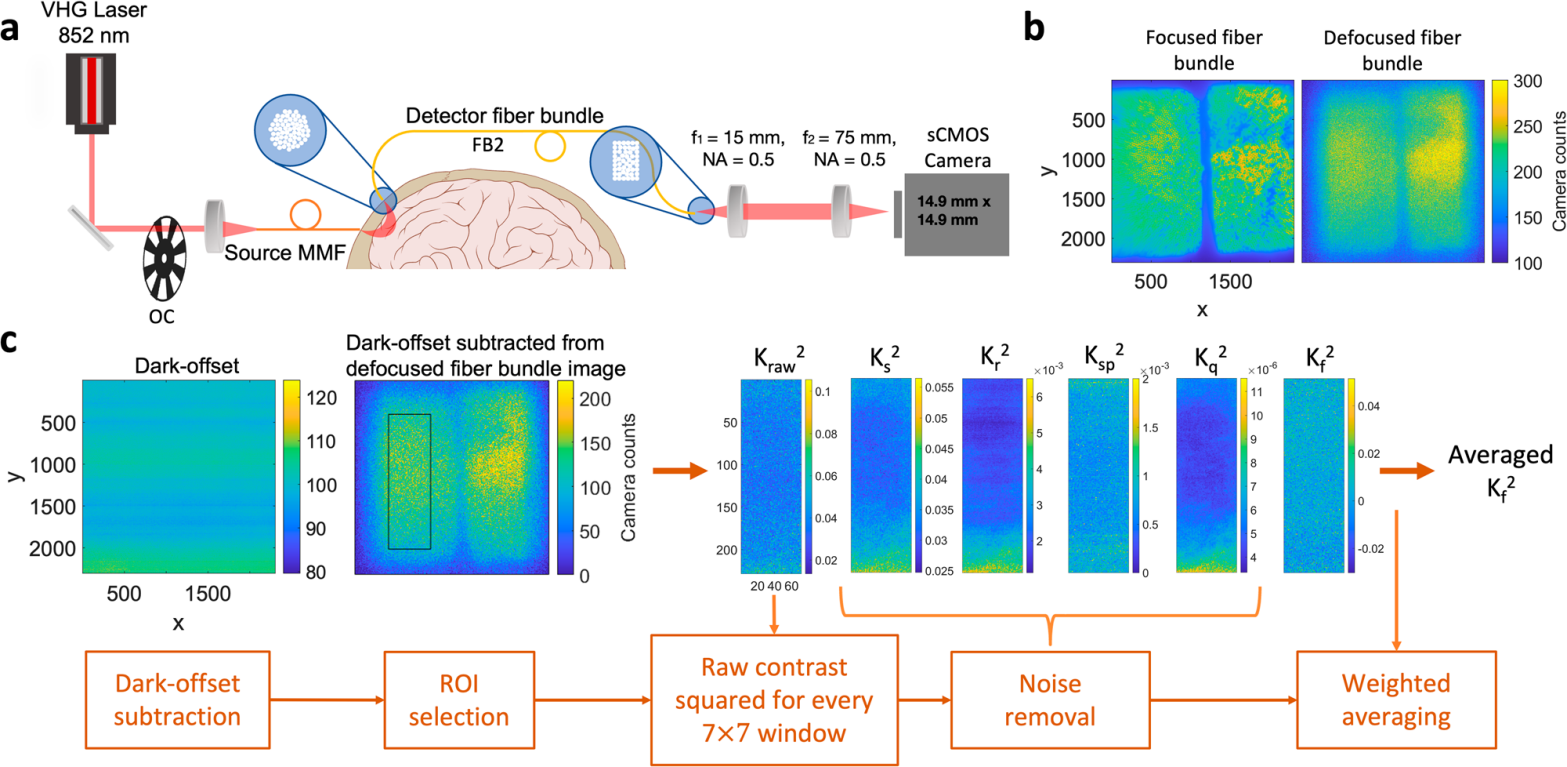
SCOS set-up and data analysis pipeline. **a** The schematic of the fiber-based SCOS set-up. Fibers are arranged into a circular bundle on the proximal end, and a rectangular bundle on the distal (camera) end. Brain and skull image is obtained from Wikimedia Commons. All the other elements of the image are generated by the authors using Microsoft PowerPoint. **b** Example speckle images when fiber bundles are focused and slightly defocused. **c** Illustration of the data analysis pipeline to remove the noise contributions from the measured ${K}_{{raw}}^{2}$ to obtain ${K}_{f}^{2}$ which is inversely proportional to *BFi*. VHG is volume holographic laser, OC is optical chopper, MMF is multi-mode fiber, FB is fiber bundle, NA is numerical aperture, CMOS is complementary metal-oxide-semiconductor, ROI is region of interest.

Schematic of the fiber-based SCOS system, example speckle images, and the illustration of the data processing pipeline are shown in Fig. 1. Coherent light was launched through the multi-mode fiber onto the forehead and the diffusely remitted light was collected by a detector fiber bundle and subsequently imaged by a sCMOS camera (Fig. 1a). To populate the camera pixels, we imaged two rectangular fiber bundles at the same SDS onto the same camera. We show two example images of the fiber bundles focused and slightly defocused in Fig. 1b. For SCOS measurements, we kept the fiber bundles slightly defocused by moving the distal end out of focus to improve the spatial uniformity of the image from the individual fibers in the bundle. The distal end of the fiber bundle is fixed once defocused to keep the same defocusing for all the measurements. The data analysis pipeline in Fig. 1c removed the bias in the raw speckle contrast, ${K}_{{raw}}^{2}={{{{\mathrm{var}}}}}(I)/{\left\langle I\right\rangle }^{2}$, that arises from shot and instrumental noise sources and measurement non-uniformities, to obtain the fundamental spatial contrast squared ${K}_{f}^{2}$ (see Methods section), where *I* is the measured speckle intensity in the unit of camera counts (ADU), *var* is the variance. Here ${K}_{f}^{2}$ is directly related to the intensity auto-correlation function ${g}_{2}\left(\tau \right)=1+\beta {e}^{-2\tau /{\tau }_{c}}$ obtained from DCS measurements^36^, where *τ* is the delay time, *τ*_*c*_ is the decorrelation time, and *β* the coherence parameter. For long camera exposure times, ${T}_{\exp }\gg {\tau }_{c}$, which is valid in most human brain measurements (${T}_{\exp } \sim 1{ms},{\tau }_{c} \sim 10\mu s$), the blood flow index *BFi* or $1/{\tau }_{c}$ is proportional $1/{{K}_{f}}^{2}$^14,22^.

**Fig. 2:**
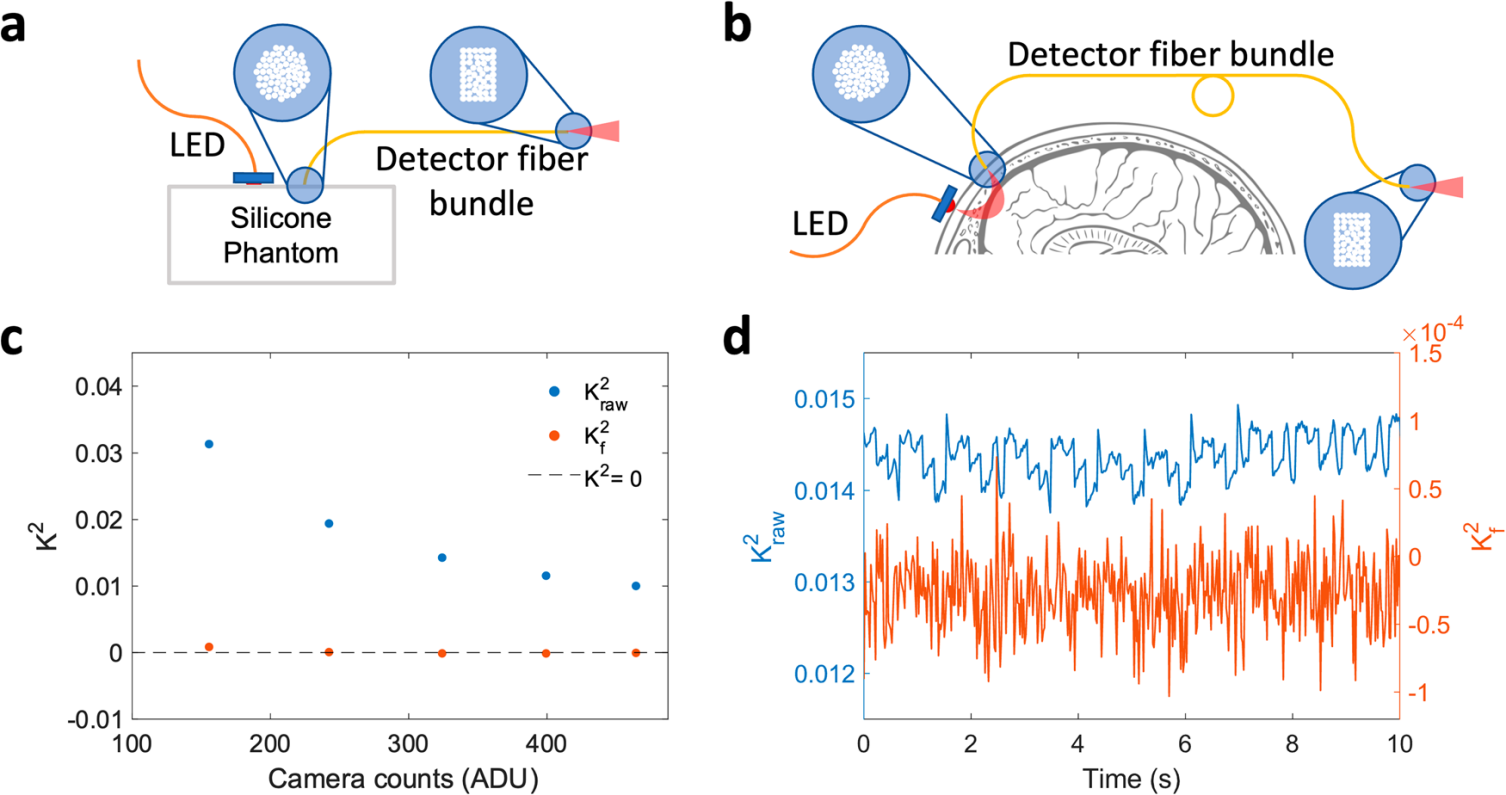
Validation of the data analysis pipeline for fiber-based SCOS. **a** Schematic experimental setup for LED measurements of the static phantom sample. The DC powered 850 nm LED is placed 15 mm away from detector fiber bundle. **b** Schematic experimental setup for human forehead LED measurements. **c** ${K}_{{raw}}^{2}$ and ${K}_{f}^{2}$ as a function of mean intensity $\left\langle I\right\rangle$ for the phantom sample LED measurements. **d** An example time course of ${K}_{{raw}}^{2}$ and ${K}_{f}^{2}$ for human forehead LED measurements.

To validate the data processing stream, we tested SCOS measurements using a light-emitting diode (LED) light source (λ = 850 nm) as illustrated in Fig. 2. Since LED light is incoherent, no speckles will be generated and ${{K}_{f}}^{2}$ is expected to be zero. But ${K}_{{raw}}^{2}$ will be non-zero because of noise and non-uniformity in illumination. The measurement schematic and the results from measurements on a dynamic phantom sample are shown in Fig. 2a, c. We found that ${K}_{{raw}}^{2}$ was approximately 0.02, reducing with increasing mean intensity because of reduced shot noise contribution to the bias, but that after correction ${{K}_{f}}^{2}$ was greatly reduced towards the expected value of 0 (i.e. $\sim {10}^{-4}-{10}^{-5}$). The system schematic and results for human forehead baseline measurements at $\rho =33$ mm are shown in Fig. 2b, d. We observed a cardiac pulse pattern in ${K}_{{raw}}^{2}$ because of cardiac pulse induced changes in the measured intensity modulating the shot-noise contribution which modulates the measured contrast. After correction, the residual ${K}_{f}^{2}$ was on the order of 10^−5^, which is two orders of magnitude smaller than the blood flow induced ${K}_{f}^{2}$ (~10^−3^) measured using coherent laser light in SCOS measurements. This illustrates the validity of the data processing stream to correct for the bias induced by noise in SCOS measurements.

**Fig. 3:**
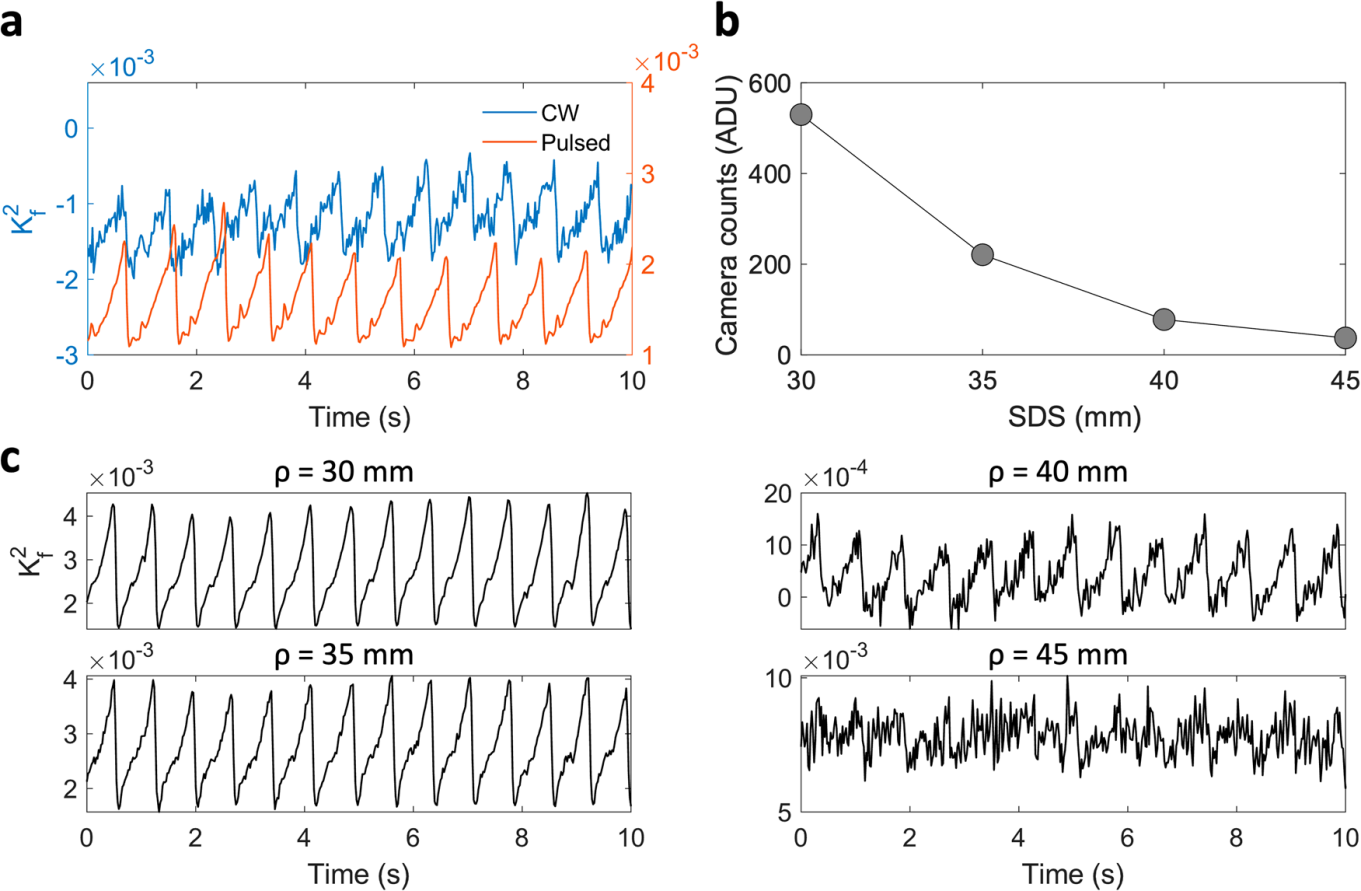
Pulsing strategy and cardiac measurements at SDS ranging from 30 to 45 mm. **a** Comparison of CW and pulsed fiber-based SCOS from baseline measurement on the human forehead at an *ρ* = 33 mm. **b** Mean camera counts and **c** time series of ${K}_{f}^{2}$ from baseline fiber-based SCOS measurements on the human forehead at SDS ranging from *ρ* = 30 to 45 mm. This is measurement was performed on a single participant. No trial averaging is used.

To improve the photon flux within a particular camera frame, we implemented a pulsing strategy with a rotating optical chopper for the laser source (Fig. 1a). The duty cycle of the optical chopper was set at 10% so we could use 10× higher photon flux per camera exposure time while keeping the average incident power as well as the peak power to be in compliance with ANSI safety standard limits at λ = 852 nm. We also demonstrated the SNR improvement by measuring cardiac signals with and without the pulsing strategy at *ρ* = 33 mm (Fig. 3a) for a qualitative comparison, with a quantitative comparison using a dynamic phantom sample shown in Supplementary Fig. 1. The lower photon flux in continuous wave (CW) mode resulted in higher instrumental noise, i.e. the measurement is in the read noise regime, leading to an inaccurate noise correction, likely because of instabilities in the camera read noise, and thus we obtained negative ${K}_{f}^{2}$ values. On the other hand, the higher photon flux in the pulsed mode overcomes the instrumental noise sources, permitting an accurate calibration to correctly estimate ${K}_{f}^{2}$ (see Supplementary Figs. 2–3). We also show examples of the measured cardiac signals at various SDSs ranging from *ρ* = 30 mm to 45 mm (Fig. 3b, c). At larger SDS, the correction for the bias became less accurate due to the instability of the read noise calibration for the camera we used. Thus, ${K}_{f}^{2}$ did not accurately report the absolute blood flow when read noise became appreciable at *ρ* = 40 mm and longer. Nevertheless, we still observed clear cardiac signal at *ρ* = 40 mm, indicating the possibility of using SCOS to measure relative blood flow changes at 40 mm or greater SDS provided a camera with a more stable read noise can be employed, or more speckles are integrated per camera pixel to maintain shot-noise performance.

**Fig. 4:**
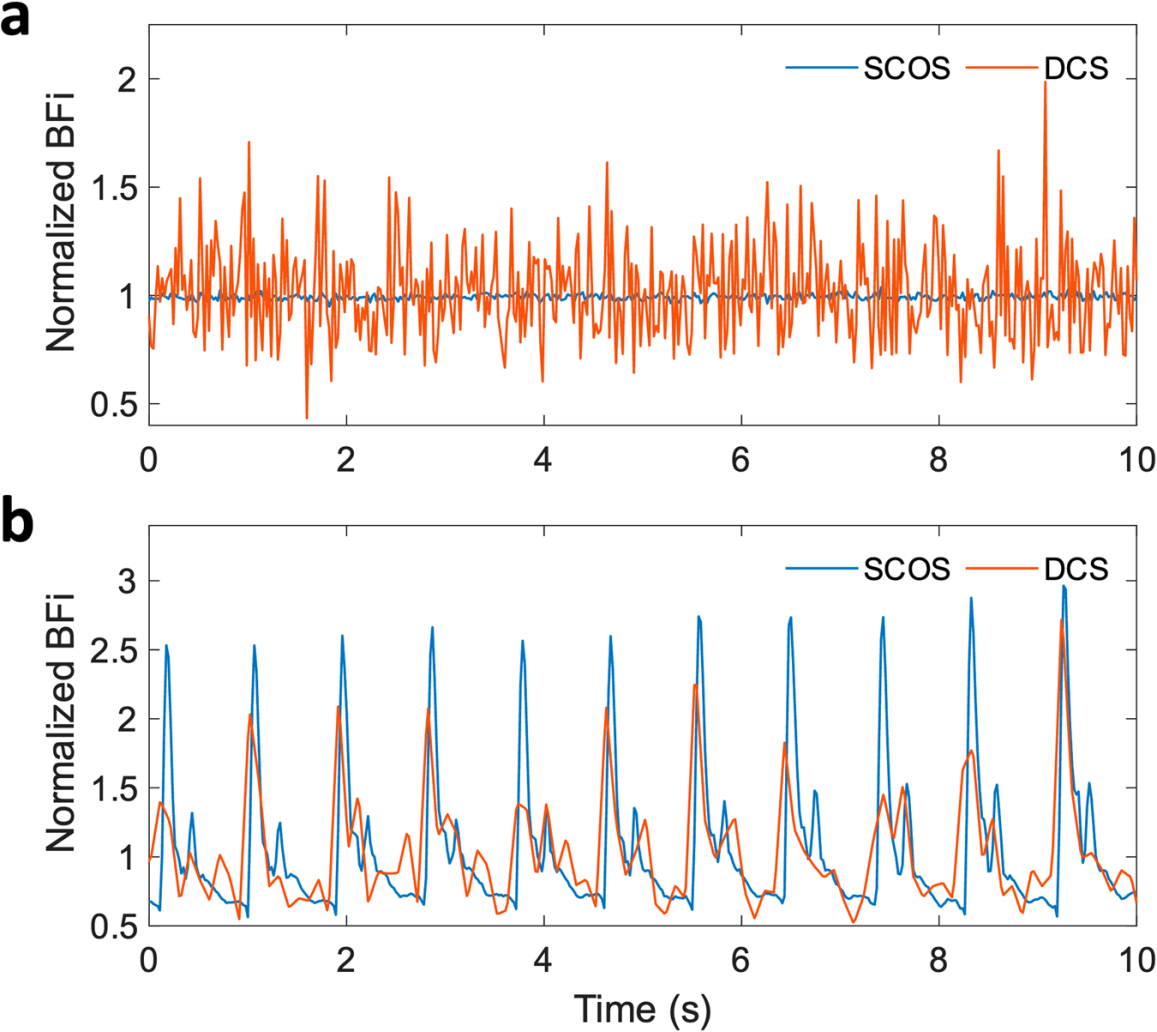
Comparison between fiber-based SCOS and DCS. **a** Normalized *BFi* of the dynamic phantom with 20 mW CW at *ρ* = 20 mm. The frame period of fiber-based SCOS and the integration period of DCS are both set at 21.7 ms. **b** Human forehead normalized BFi with 35 mW CW at *ρ* = 20 mm. SCOS at 46 Hz (21.7 ms frame period) and DCS at 100 ms integration time.

Next, we compare the performance of SCOS with a state-of-the-art single-channel DCS system (Excelitas SPAD + PicoHarp time tagger) using the same CW light source. We first compared the measurements using a liquid dynamic phantom (intralipid 1.6% v/v in deionized water at room temperature) at *ρ* = 20 mm (Fig. 4a). Both the SCOS camera’s frame period and DCS g_2_(τ) integration time were set at 21.7 ms. 71.4% of the camera pixels were used for SCOS analysis, focusing on areas of high illumination from the fiber bundles. SNR was calculated as ${K}_{f}^{2}/{std}({K}_{f}^{2})$ and ${\tau }_{c}/{std}({\tau }_{c})$ for SCOS and DCS measurements respectively, where *std* is the standard deviation. The SNR for DCS and SCOS was 5 and 114.8 respectively, representing a 23 fold increase afforded by SCOS. If the camera pixels were fully illuminated, we expect a further 18% increase resulting in a 27 fold SNR improvement of SCOS versus DCS.

We demonstrated this SCOS improvement in SNR over DCS qualitatively through measurements of cardiac signals on the human forehead using SCOS and DCS at a short SDS of *ρ* = 20 mm (Fig. 4b). Since the SNR for DCS is lower, we used a longer integration time of 100 ms for DCS to compare the signal quality with that of SCOS. Here *BFi* is calculated as $1/{{K}_{f}}^{2}$ for SCOS and $1/{\tau }_{c}$ for DCS. Despite the longer integration time for DCS, SCOS still has higher signal quality, which is apparent from the distinct features of the systolic peak and dicrotic notch in every pulse (Fig. 4b). This is consistent with the greater than order of magnitude difference in SNR observed with dynamic phantom samples.

**Fig. 5:**
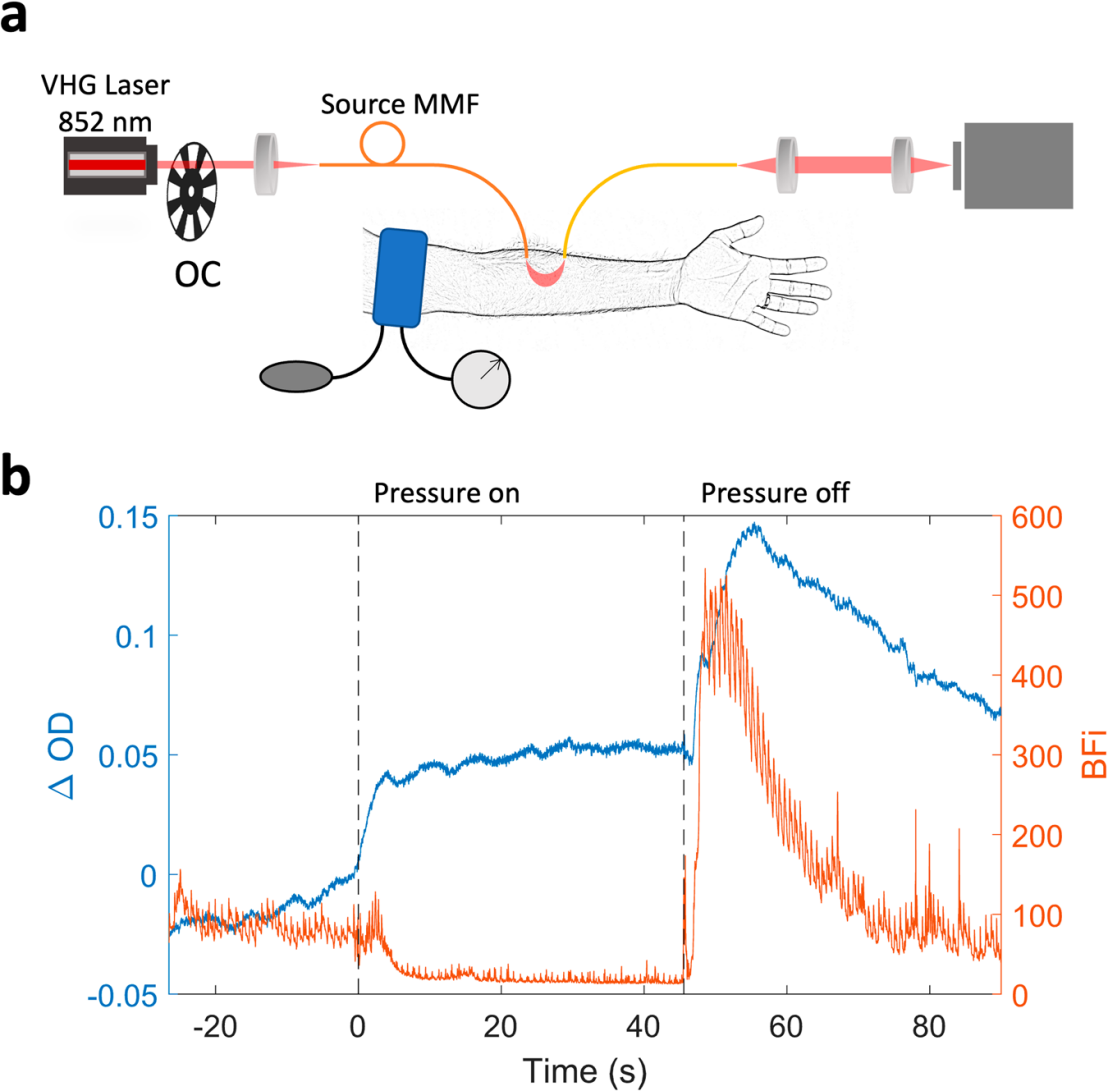
Cuff occlusion measurements using fiber-based SCOS. **a** Schematic representation of the cuff-occlusion measurements. Extended arm image is obtained from Wikimedia Commons. All the other elements of this image are generated by the authors using Microsoft PowerPoint. **b** The time courses of BFi and ∆*OD* for cuff-occlusion measurements.

In Fig. 5, we show the fiber-based SCOS measurement of blood flow and change in optical density (∆*OD*) during a cuff-occlusion measurement on the human forearm (Fig. 5a). ∆*OD* is defined as $\triangle {OD}={\log }_{10}({I}_{0}/I(t))$, where *I*_0_ is the intensity at the baseline. When the applied external pressure is greater than systolic pressure, both the arteries and the veins were occluded. As expected, the *BFi* decreased during occlusion and there was a reduction in the ~1 Hz cardiac fluctuations^20,37^. As seen in Fig. 5b, the optical density increased since during the first few seconds of occlusion, the applied pressure only occludes the veins and arterial inflowing blood increases the blood volume, and thus total hemoglobin concentration. When the applied external pressure was released, both *BFi* and ∆*OD* overshot due to the classic hyperemic overshoot to supply the oxygen starved tissue with oxygen, followed by a gradual recovery to baseline levels.

**Fig. 6:**
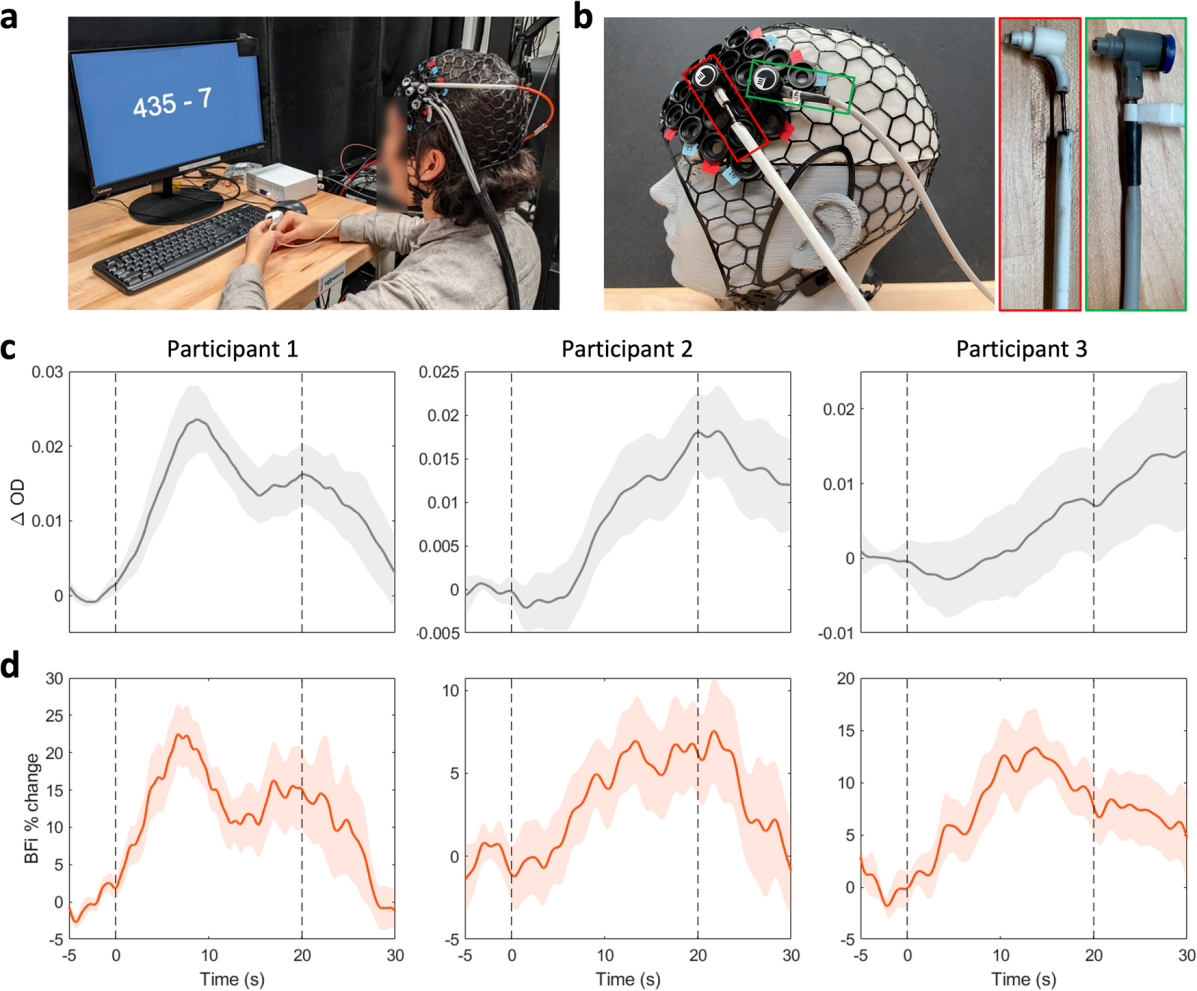
Mental subtraction measurements using fiber-based SCOS. **a** Example of the mental subtraction task conducted by a subject. **b** Placement of the NinjaCap and fibers on a participant’s head represented by the mannequin head. Source fiber (in red) and detector fiber bundle (in green) on a NinjaCap with NIRx spring loaded grommets. **c** Trial averaged (*n* = 15) OD change from baseline (t = −5 to 0 s) resulting from the mental subtraction task (presented from t = 0 s to t = 20 s). The shaded area indicates the standard error of the trial averaged response. **d** Trial averaged change in BFi from baseline. For both BFi and ∆*OD*, one ROI with clear trial averaged response was selected.

Finally, we show human brain function measurements during a mental subtraction task at *ρ* = 33 mm for three participants in Fig. 6. As described in the Methods section, we first used our existing high-density fNIRS system to locate the activation region on the forehead with the largest task-averaged change in total hemoglobin concentration. We then placed the fiber-based SCOS source and detector optodes in the same region to measure changes in CBF. Of the five participants measured for SCOS, two did not show change in signal intensity (equivalent to single wavelength fNIRS) with mental subtraction. Since the same mental subtraction task (with different numbers) was used in the fNIRS measurements, it is possible that these participants had already developed different strategies for the math problems thus not showing activation in SCOS measurements. We only used the ROI with sufficient trial-averaged response in ∆*OD* to calculate trial-averaged response in BFi. We found that for all participants with sufficient ∆*OD* during mental subtraction (Fig. 6c), blood flow significantly increased by 7–16% during activation (p = 7.7E-8, 0.0083, 2.1E-4 for participant 1, 2, 3 respectively) (Fig. 6d) and returned to baseline values post-activation, consistent with the expected values from the literature^38,39^. This work demonstrates the promising capabilities of SCOS for human brain function measurements.

## Discussion

We have demonstrated a measurement of human CBF and brain function using a fiber-based SCOS system with a CMOS sensor. We have developed and validated the data analysis pipeline to correct for noise-induced bias terms in the measured ${K}_{{raw}}^{2}$ typically ignored in mouse brain LSCI measurements due to higher photon flux^20,21^. With SCOS, shot-noise limited measurements can be achieved without employing interferometric detection, as has been done previously for DCS^19^ and SCOS^18^, by adjusting the measurement parameters including the speckle-to-pixel size ratio and the camera exposure time^34^. However, the integration of fiber-based SCOS (this work) with interferometry could be advantageous for measurements that desire a reduced camera integration time or have less photons per camera frame, or when using a camera with higher read noise. At the photon flux achieved with *ρ* = 33 mm on the human forehead, we believe there is little value in integrating interferometry to our fiber-based SCOS system as we are able to achieve shot-noise performance without interferometry.

In this manuscript, we have demonstrated the validity of our noise correction scheme for achieving shot-noise limited measurements. Using our recent SCOS noise model^34^, we found that we could achieve a shot noise limited measurement of speckle contrast when the camera count is above ~100 ADU per pixel for our Hamamatsu camera. We used a pulsing strategy and low speckle-to-pixel ratio (s/p < 1) to achieve 200 ADU and greater camera counts for our human brain measurements at *ρ* = 33 mm (Fig. 2c). At lower camera count levels, our correction scheme was challenged mainly due to temporal variation in read noise for our camera (Supplementary Fig. 2). While keeping our measurements in the shot-noise limited regime, we improved noise correction by measuring dark images and estimating the read noise pattern before each measurement session. Additionally, by exploiting the use of a large number of pixels on the CMOS camera and utilizing the laser pulsing strategy, our fiber-based SCOS system can measure pulsatile *BFi* from the human forehead at up to *ρ* = 40 mm. At *ρ* = 33 mm, the high brain-to-scalp sensitivity greatly improved brain function measurements^19^ over prior measurements at 20 mm^25^. The sensitivity to the brain signal can be further improved using data analysis methods such as short separation regression^40^ or multi-layer modeling of CBF^41^ to disentangle brain signals from scalp signals.

### Cost consideration

In the results section, we compared the performance of our fiber-based SCOS with a single channel DCS system. We have mentioned before an SNR improvement of 23 fold with SCOS over DCS, and an expectation of a 27 fold SNR improvement with full coverage of the camera. With cost taken into account, our fiber-based SCOS system (with an sCMOS camera) can achieve a 13.5× improvement in SNR of CBF estimation compared to a traditional DCS system (with a SPAD detector) with a similar cost (i.e. by increasing the DCS channel count to 4). Note that we compared the performance of DCS and SCOS experimentally using CW light sources, since we do not have a DCS system with pulsed laser (and there is no need to implement such system). When the light source is pulsed at 10% duty cycle, the peak photon flux is increased by 10×. For SCOS the measurement rate is not impacted since it is limited by the camera frame rate (our modeling results show that the SNR versus *T*_*exp*_ plateaus at around ~2 ms exposure time, so that there is dead time between camera frames that can be utilized for pulsing), such that we can achieve close to ~10× improvement in SNR (Supplementary Fig. 1). For DCS, the measurement rate or integration time is reduced to 10%. Therefore, the improvement of SNR in pulsed SCOS will be larger than that of pulsed DCS. Our modeling work has demonstrated ~2.4× improvement of SNR for pulsed DCS versus CW DCS (Supplementary Note 1). This results in an SNR improvement of ~56x for pulsed SCOS over pulsed DCS with a similar cost. The cost of a single channel SPAD detector is >$3k while the cost of a sCMOS camera is >$10k. For applications with less stringent SNR requirements, there are options for even lower cost CMOS cameras at the expense of higher read noise, lower bit depth, and potentially non-linear and non-uniform camera gain across pixels. For example, we carried out a preliminary measurement of the cardiac signal on the human forehead at *ρ* = 33 mm using a low-cost CMOS camera (Basler acA1920-160umPRO), which shows a promising high signal quality (Supplementary Fig. 4). While this is beyond the scope of the current manuscript, we believe it is important in the future to characterize different camera options that could be suitable and cost-effective for different applications of SCOS systems. Apart from the cost consideration, we found that the photon flux per speckle in our SCOS system is about 9 times smaller than that of the DCS. Some contributing factors include the energy loss in the lens system and the lower coupling efficiency of higher-order modes in multi-mode fibers. Future work could look into improving the optical design to narrow this gap to achieve even better performance from SCOS systems.

## Methods

### Fiber-based SCOS system

The schematic of our fiber-based SCOS system is illustrated in Fig. 1. The input laser light source (Thorlabs free-space VHG, 852 nm) is coupled into a multi-mode fiber (200 *μ*m core diameter, 0.5 NA) which is coupled to an acrylic light pipe of 3.5 mm diameter that delivers the light to a sample such as the human forehead. We utilized custom-made fiber bundles (~3770 strands of 37 µm core diameter multimode fiber, 0.66 NA) with circular shape on the proximal end (~2.5 mm diameter) to facilitate scalp contact through the hair and rectangular shape on the distal end (~1.64 × 3 mm) to match the shape of the CMOS sensor as detectors and imaged two fiber bundles from different locations on the human scalp at the same SDS onto a single sCMOS (Hamamatsu, Orca Fusion BT) camera. The lenses form a 4 f system as illustrated in Fig. 1a. The camera operates with the default settings of 16-bit resolution, fast scan operating mode with an expected read noise of 1.4 e^−^ across the sensor. We slightly defocus the imaged speckle to fill the gaps between individual fibers within a single fiber bundle as shown in Fig. 1b. The speckle to pixel size ratio (s/p) for the system has been calibrated to be 0.57 from ${K}_{f}^{2}=\frac{1}{2}{(s/p)}^{2}/(1+{(s/p)}^{2})$ for unpolarized light obtained using a static phantom sample^34^ at high photon flux values to reduce errors introduced by noise.

### Image acquisition software

A custom data acquisition software was built on C++ and Python using DCAM-API 4.1 to interface with the sCMOS camera and PH300 driver to interface with the photon counting module (PicoHarp 300) connected to a SPAD (SPCM-AQ4C, Excelitas). Both SCOS and DCS measurements can be simultaneously performed by running two instances of the software. The sCMOS camera, SPAD and the laser source optical chopper are externally triggered for synchronization. Depending on the device utilized, either the raw sCMOS camera images or the SPAD timestamps are sent to a data acquisition computer (AMD Ryzen Threadripper 3970x CPU, NVIDIA Quadro RTX 4000 GPU, 64 GB of 2666 MHz RAM) via two Camera Link cables using the Camera Link serial communication protocol and saved to a local NVME SSD hard drive in hierarchical data format 5.

### Data analysis pipeline for fiber-based SCOS

Raw data of intensity patterns for all the camera frames are recorded. The data processing stream is shown in Fig. 1c. We first subtract the raw intensity pattern by the dark offset which is obtained as the average of 500 dark images taken when the laser light is turned off. We then select the region of interest (ROI) for the speckle contrast calculation. Raw contrast squared is calculated as ${{K}_{{raw}}}^{2}=({std}{(I)/\left\langle I\right\rangle })^{2}$ for each sliding window composed of 7 × 7 pixels within the ROI, where the intensity *I* is measured in ADU for all the pixels. We have used linear fitting to reduce the noise arising from estimation of $\left\langle I\right\rangle$ for each window. We first obtain the average intensity for all the pixels as a function of time denoted as ${I}_{{all}}(t)$. We assume that the temporal shape of the average intensity for each window denoted as ${I}_{w}(t)$ is linearly related to ${I}_{{all}}(t)$ as ${I}_{w}\left(t\right)={a* I}_{{all}}\left(t\right)+b$. The coefficients *a* and *b* are obtained from fitting and $\left\langle I(t)\right\rangle ={a* I}_{{all}}\left(t\right)+b$, which is a smoothed version of ${I}_{w}\left(t\right)$, is used as the denominator to calculate *K*_*raw*_ for each window. Examples of ${I}_{{all}}(t)$, ${I}_{w}\left(t\right)$, and ${a* I}_{{all}}\left(t\right)+b$ are shown in the Supplementary Fig. 5. After obtaining the raw contrast squared ${{K}_{{raw}}}^{2}$, we remove the bias induced by shot noise ${K}_{s}^{2}$, read noise ${K}_{r}^{2}$, spatial non-uniformity in illumination ${K}_{{sp}}^{2}$, and quantization error ${K}_{q}^{2}$ as

1 $${K}_{f}^{2}={K}_{{raw}}^{2}-{K}_{s}^{2}-{K}_{r}^{2}-{K}_{{sp}}^{2}-{K}_{q}^{2},{K}_{s}^{2}=\frac{g}{\left\langle I\right\rangle },{K}_{r}^{2}=\frac{{\sigma }_{r}^{2}}{{\left\langle I\right\rangle }^{2}},{K}_{{sp}}^{2}=\frac{{\sigma }_{{sp}}^{2}}{{\left\langle I\right\rangle }^{2}},{K}_{q}^{2}=\frac{1}{12{\left\langle I\right\rangle }^{2}},$$

where *g* is the camera gain, *σ*_*r*_ is the read noise of the cameras, ${\sigma }_{{sp}}$ is the variance obtained from the temporally averaged speckle image over 500 camera frames. ${K}_{q}^{2}$ comes from the digital format of the camera output, in which a step size of one adds variance of 1/12^42^. To avoid erroneous impact of noise sources, the pixels with high read noise value i.e. σ_r_ > 10 and hot pixels of the camera are ignored. This correction process is also done for each 7 × 7 pixels. We then perform a weighted average of ${K}_{f}^{2}$ by *I*^2^ for all the windows within the ROI to obtain a single value of ${K}_{f}^{2}$ for a camera frame. We calculate ${K}_{f}^{2}$ for all the camera frames to obtain the time course of ${K}_{f}^{2}\left(t\right).$ The average intensity ${I}_{{all}}\left(t\right)$, simplified as $I\left(t\right)$, is also obtained for all the camera frames. ${K}_{f}^{2}$ is related to the decorrelation time of *τ*_*c*_ and exposure time of *T*_*exp*_ via^21,22^

2 $${K}_{f}^{2}=\beta \frac{{\tau }_{c}}{{T}_{\exp }}\left[1+\frac{{\tau }_{c}}{2{T}_{\exp }}\left({{\exp }}\left(\frac{-2{T}_{\exp }}{{\tau }_{c}}\right)-1\right)\right]$$

### Pulsing strategy to improve photon flux

We have utilized a pulsing strategy to improve the photon flux received within a camera frame. A rotating chopper wheel with 10% duty cycle is implemented to generate pulsed light to increase the photon flux within the camera exposure time while keeping the average incident power at 33 mW, within the ANSI safety limit at *λ* = 852 nm. The pulsed laser is synchronized with the camera frames during measurements by phase-locking the chopper wheel with the camera exposure sync signal, and as a safety precaution a 13 Hz trigger signal was utilized in place of the camera exposure sync signal when the camera was not exposing frames to ensure the light is pulsed while not performing measurements.

### DCS system and data analysis

We use a DCS system consisting of a single channel SPAD (SPCM-AQ4C, Excelitas) with a time tagger (PicoHarp 300, PicoQuant). The source fiber and CW laser are shared with the fiber-based SCOS system. The DCS system is synchronized with the fiber-based SCOS for simultaneous data acquisition. The intensity temporal autocorrelation function (${g}_{2}\left(\tau \right)$) was generated from the raw intensity counts for time delay from 1 µs to 14.3 ms, with exponential increase in step between time delays. The bin size was set at 1 µs for the measurement time of 100 ms. The laser coherence (β) was estimated from fitting the ${g}_{2}\left(\tau \right)$ curve averaged over the duration of the measurement to an exponential decay function given by^36,37^:

3 $${g}_{2}\left(\tau \right)=1+\beta {{\exp }}\left(\frac{-2\tau }{{\tau }_{c}}\right)$$

where τ is the time delay, and *τ*_*c*_ is the decorrelation time. For each g_2_(τ) curve averaged over the measurement time the same exponential decay function and prior estimate of laser coherence is used to estimate the decorrelation time. The least-square curve fit was done using MATLAB’s lsqcurvefit function with implementation of the Levenberg-Marquardt algorithm.

### Dynamic phantom SNR measurement

The dynamic phantom was fabricated as 8% v/v ratio of 20% Intralipid (batch 10LI4282, Fresenius Kabi, Germany) in deionized water at room temperature. A 3D-printed mount made of flexible material (NinjaFlex TPU filament, NinjaTek) was used to mount the fiber-based SCOS fiber bundle and DCS single mode fiber at *ρ* = 20 mm from the source fiber.

### fNIRS and fiber-based SCOS brain activation data acquisition

To better localize the brain activation region, we utilize the high-density fNIRS system with a commercially available NIRSport2 (NIRx) with 14 sources and 32 detectors on the prefrontal cortex region with first and second nearest neighbor separation distances of 19 and 33 mm respectively. The sources and detectors are placed in a NinjaCap, an in-house 3D printed fNIRS cap made of flexible material (NinjaFlex, NinjaTek) with NIRx grommets for compatibility with variable tension spring holders. The cap is placed on the head with respect to EEG 10–10 midline central electrode site (Cz), which is estimated through measurement of nasion, inion, left/right pre-auricular points. The signal quality for each source-detector pair is tested through the Aurora fNIRS acquisition software (NIRx). fNIRS measurement during mental subtraction task is also done using the Aurora fNIRS acquisition software. The fNIRS data is analyzed with Homer3^43^ to obtain trial averaged oxyhemoglobin (HbO), deoxyhemoglobin (HbR), and total hemoglobin (HbT) changes for all source-detector pairs. The source-detector with largest HbO and HbT change is selected for subsequent fiber-based SCOS measurement.

For SCOS measurements, the same NinjaCap used in fNIRS measurements is placed on the participant’s head, followed by placement of custom source fiber and detector fiber bundle at the pre-selected source-detector pair. After baseline measurement for confirmation of photon flux, the participant undergoes mental subtraction task. The externally triggered camera sends images via Camera Link cable and card to the computer where the images are written to hard drive for processing.

### Cuff-occlusion and mental subtraction task

A sphygmomanometer with an analog manometer and a manually operated bulb was used to apply cuff occlusion. The inflatable cuff was placed around the left arm. The fiber-based SCOS source and detector fibers were placed on the left ventral forearm at *ρ* = 30 mm. The participant was optically measured for 120 s, consisting of 30 s of baseline, 45 s of cuff occlusion, and 45 s of return to baseline. For cuff occlusion, the bulb was squeezed repeatedly to increase the pressure to 180–200 mmHg within five seconds. The pressure was kept above 180 mmHg until pressure release happening over less than five seconds (Fig. 5).

During the mental subtraction task, the participants are seated in front of a computer monitor providing mental subtraction task stimulus. After the baseline measurement, the participant is visually presented with a random selection of a three-digit number and a smaller number (6, 7, or 13) in a mathematical expression format (e.g. 270 – 7). The participant then serially subtracts the smaller number from the larger number (e.g. 270, 263, 256) until the problem disappears from the screen. Each problem is displayed for 20 s and the interval between problems is randomly selected from 10 to 20 s. Each measurement consists of five trials, with three measurements totaling 15 trials per participant for a total duration of 12 min.

### Mental subtraction task specific data analysis

The fNIRS data is analyzed using a processing stream in Homer3. The processing stream consists of (1) pruning channels with a SNR threshold of 10, (2) converting intensity values to change in OD, (3) applying a motion detection and correction algorithm^38^, (4) bandpass filtering the data from 0.01 to 0.5 Hz to remove signal drift and cardiac fluctuations, (5) converting change in OD to changes in HbO and HbR concentration using modified Beer-lambert law^39^, and (6) applying a general linear model (GLM) with signal from *ρ* = 19 mm.

From the raw SCOS data, the entire data analysis pipeline is utilized to remove the noise contribution and to obtain BFi. Next, periodic cardiac fluctuations are removed from BFi through a cardiac signal removal algorithm. The algorithm identifies the systolic peaks in the BFi to obtain the mean cardiac signal. With the assumption that each cardiac signal is of similar shape, the mean cardiac signal is linearly fitted to individual cardiac signal using the least squares method and subtracted to remove the cardiac signal contribution. Any residual signal is removed through a 13th order median filter and a third order Butterworth low-pass filter at 0.5 Hz. Same processing is implemented for intensity time course to get cardiac-free ∆*OD*. The trial averaged ∆*OD* time course is obtained for each ROI. The ROI with sufficient trial averaged ∆*OD* response is used to obtain the trial averaged BFi response.

### Participants

Eight participants within 20 to 60 years age group with no prior diagnosis or treatment of neurological disorders were recruited for this study. Sex, gender, race, and ethnicity were not considered during recruitment. Participants were recruited through on-campus advertisements including flyers. Seven participants were recruited for measurement of mental subtraction induced CBF changes, and one participant was recruited for the cuff occlusion measurement. One participant performed both mental subtraction and the comparison of cardiac signals between DCS and fiber-based SCOS, with and without pulsing strategy, and fiber-based SCOS over a range of SDSs. Of the seven participants for mental subtraction task, five showed lateral frontal lobe activation and thus were subsequently imaged for fiber-based SCOS measurements. The experimental procedure and protocols were approved and carried out in accordance with the regulations of Institutional Review Board of the Boston University. Each participant provided a signed written informed consent form prior to the experiment.

### Statistics and reproducibility

SNR is defined as the ratio between mean and standard deviation of the BFi time course when comparing SCOS and DCS. For the interval plot in Fig. 6, the solid line represents the mean response averaged over 15 trials for each participant. The error bars are standard error for each trial-averaged data point. Normal distribution across trials was assumed to calculate standard deviation and standard error for each trial-averaged data point. Peak of each participant’s trial-averaged response within the 20 s of stimulation and its corresponding standard error was selected to calculate the p-value (Fig. 6d)^44^ with the significance level set to *p* = 0.05. The mean, standard deviation and standard error are estimated from built-in MATLAB functions.

### Reporting summary

Further information on research design is available in the Nature Portfolio Reporting Summary linked to this article.

### Supplementary information

Supplementary Information

Reporting Summary

## Data Availability

Data used to generate the main and supplementary figures are available on Figshare database (10.6084/m9.figshare.23811402). All other datasets that support the findings of this study are available from the corresponding author upon reasonable request.

## Code availability

The SCOS data analysis pipeline is written in MATLAB. The code is available on GitHub (https://github.com/BUNPC/2023-SCOS) and has been deposited to Zenodo (https://zenodo.org/record/8206356). Any other code including those used to generate the manuscript’s figures are available from the corresponding author on reasonable request.
