# Supplementary material for: Measuring human cerebral blood flow and brain function with fiber-based speckle contrast optical spectroscopy system
Source: Commun Biol. 2023 Aug 14;6:844. doi: 10.1038/s42003-023-05211-4 (PMC10425329; doi:10.1038/s42003-023-05211-4)
Supplement: Supplementary file 1 — Supplementary Information [file 42003_2023_5211_MOESM1_ESM.pdf]

Supplementary Information

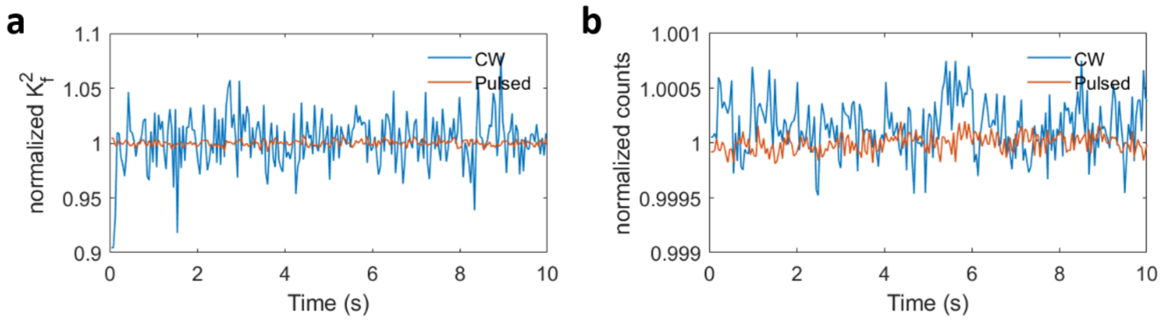

Supplementary Figure 1. Measurement of K_f^2 and intensity SNR with and without pulsing strategy. (a) Normalized fundamental contrast squared and (b) normalized camera counts from dynamic phantom at continuous and pulsed transmission (10x difference in mean intensity). We investigated the improvement in SNR of fundamental speckle contrast squared when pulsing strategy is employed. We used two different source-detector separations to change the mean intensity 10 fold. SNR was calculated as the ratio of mean value to the standard deviation of the signal remaining after 5 Hz high pass 3rd order Butterworth filter. We obtained a 3.2-fold improvement in intensity SNR and 9.2-fold improvement in K_f^2 SNR.

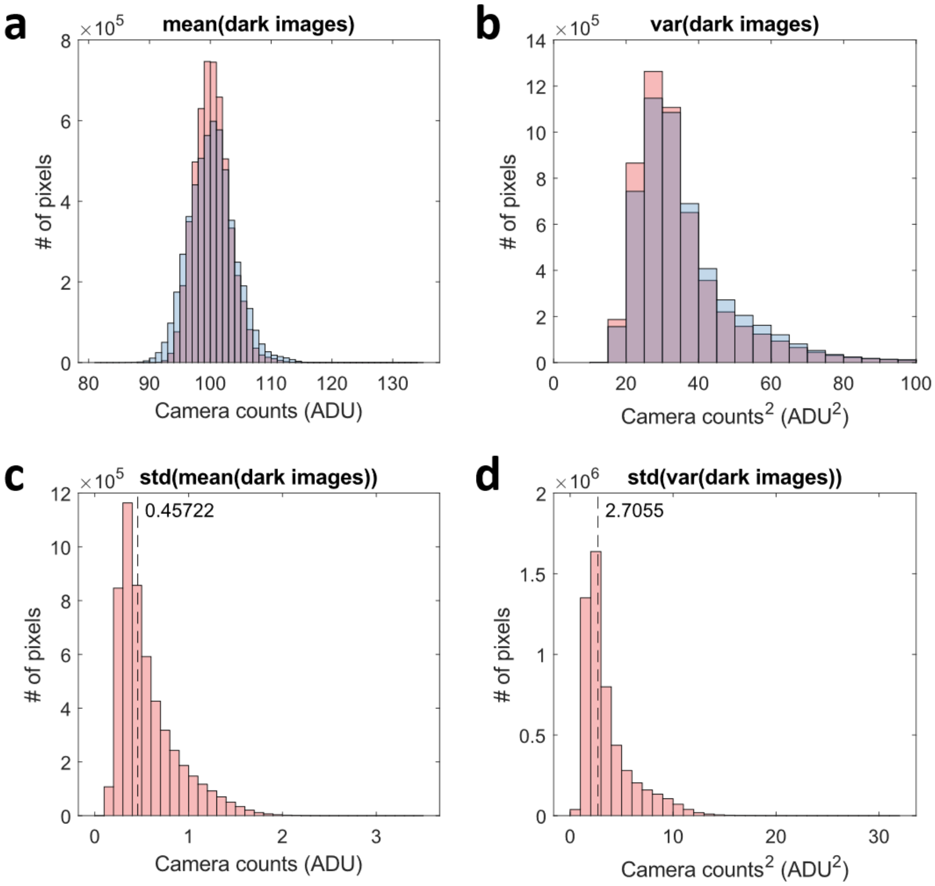

Supplementary Figure 2. Dark images to obtain read noise and dark offset. Histogram of the (a) dark offset obtained from the average of 500 dark images and (b) read noise squared (σ_r^2) obtained from the variance of 500 dark images taken 10 minutes apart. Histogram of the (c) standard deviation of the pixel-by-pixel dark offset and (d) standard deviation of the pixel-by-pixel variance of dark images across multiple measurements on same day (n = 13). We show the distribution of dark offset and read noise for all the pixels obtained from the standard deviation of the dark images on two different days. We see that the read noise distribution slightly differ and this variation can induce variations of K_r^2 on the order of 10^{-3} (close to K_f^2) for ~100 ADU. To minimize the error, we always measure the dark images before an experiment and use the dark offset and read noise for a particular measurement session.

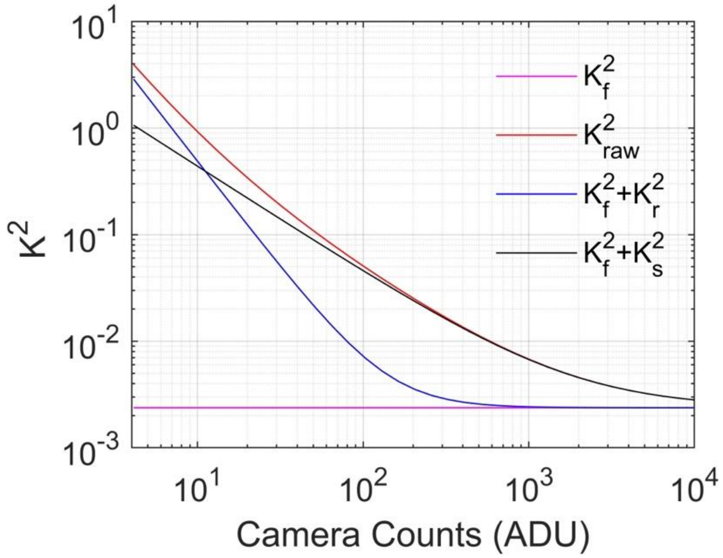

Supplementary Figure 3. The shot noise and read noise regime. K_s^2, K_r^2, K_{raw}^2, K_f^2 as functions of camera counts (ADU) with the parameters the same as in the experiments described in the Method section. We have numerically calculated the contribution of shot noise K_s^2 and read noise contribution K_r^2 to K_{raw}^2 as functions of camera counts (ADU) matching the experimental parameters described in the Methods section, using our previously established SCOS noise model in Fig. S3. We see that for signals <100 ADU, the contribution of K_s^2 is smaller than K_r^2 (i.e. the measurement is in the read noise regime), making the noise correction scheme more susceptible to temporal instabilities in read noise shown in Fig. S2 and outputting inaccurate estimation of K_f^2. For human brain function measurements, the camera counts are ~200 ADU, where K_s^2 dominates (i.e. the shot noise regime) and the noise correction scheme is robust. Also, we see that for the shot noise contribution to become negligible, i.e., $K_{raw}^2 \sim K_f^2$, that the camera counts need to be $> 10^4$ ADU, which in general is not achievable for human brain measurements. This further confirms that the noise correction is necessary for human brain measurements using SCOS.

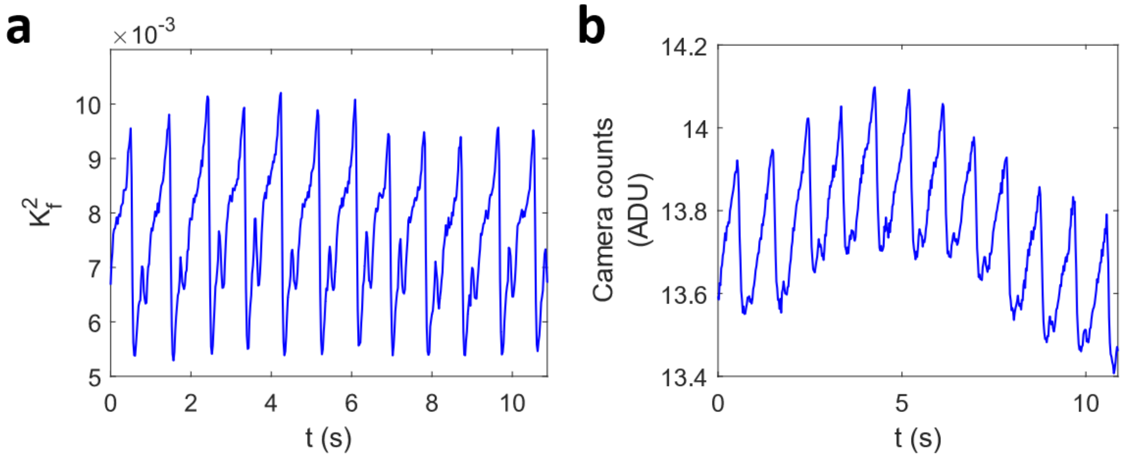

Supplementary Figure 4. Preliminary Basler cardiac measurements. (a) Fundamental contrast squared and (b) camera counts from the Basler camera baseline human forehead measurement at ρ = 33 mm showing cardiac fluctuations. We made a preliminary investigation into the use of a more cost-effective CMOS camera (Basler a2A1920-160umPRO) by measuring baseline cardiac signal with the same pulsed laser source set-up at ρ = 33 mm. The camera was operated with 8-bit depth resolution, a frame rate of 46 Hz with estimated read noise of 1.6 e- and gain of 0.37 ADU/e-.

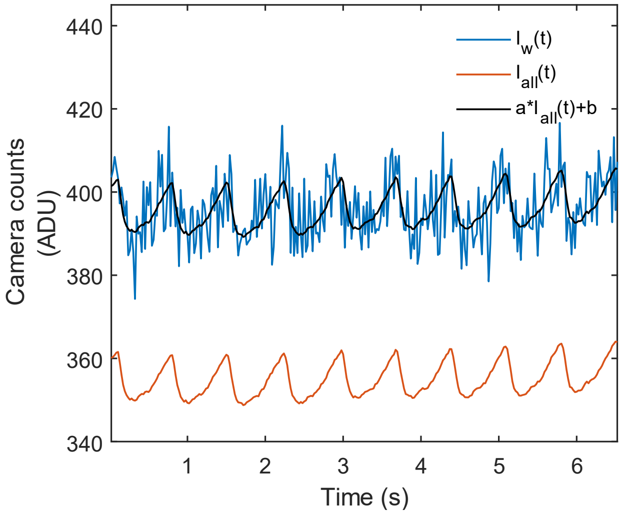

Supplementary Figure 5. Linear estimation of window $\langle I(t) \rangle$ in the contrast measurement. Estimation of window mean intensity with linear fitting. Time series of mean camera counts for a 7 x 7 pixel window (blue) and for a 500 x 1500 pixel region of interest (ROI, red). The ROI time series is linearly fitted to the window time series for a less noisy estimate of window mean intensity time series (black). As mentioned in the Methods section, we have calculated the contrast ${K_{raw}}^2 = (std(I)/\langle I \rangle)^2$ within each 7×7 window. To reduce the noise in $\langle I \rangle$, we have smoothed the average intensity within each window as described in the Methods. Examples of $I_{all}(t)$, $I_w(t)$, and $a * I_{all}(t) + b$ are shown.

Supplementary Note 1: Improvement of the performance of DCS with the pulsing strategy

We investigated the benefits of pulsing strategy in DCS numerically. The noise model[1] used has been derived, and the calculation of SNR using the noise model has been demonstrated[2]. In short, we have utilized photon counts per speckle per μs as 0.0251 and 0.2512 for CW and pulsed mode respectively, estimated from our experimental human brain measurements at ρ =33 mm. For the SNR calculation, 10^5 iterations of noisy $g_2(\tau)$ curves were simulated and the corresponding $\tau_c s$ were calculated for CW and pulsed mode respectively. The SNR, defined as the mean divided by the std of the $\tau_c s$, are calculated. The improvement was ~2.4x for pulsed versus CW DCS, in contrast with the ~10x improvement for pulsed versus CW SCOS.

Supplementary References:

1. Zhou, C. *et al.* Diffuse optical correlation tomography of cerebral blood flow during cortical spreading depression in rat brain. *Opt. Express* **14**, 1125–1144 (2006).
2. Cheng, X., Sie, E. J., Naufel, S., Boas, D. A. & Marsili, F. Measuring neuronal activity with diffuse correlation spectroscopy: a theoretical investigation. *Neurophotonics* **8**, 035004 (2021).
