# Supplementary material for: Measuring human cerebral blood flow and brain function with fiber-based speckle contrast optical spectroscopy system
Source: Commun Biol. 2023 Aug 14;6:844. doi: 10.1038/s42003-023-05211-4 (PMC10425329; doi:10.1038/s42003-023-05211-4)
Supplement: Supplementary file 2 — Reporting Summary [file 42003_2023_5211_MOESM2_ESM.pdf]

nature portfolio

Corresponding author(s): DAPR, COMMSBIO-23-1065B

Last updated by author(s): Jul 29, 2023

Reporting Summary

Nature Portfolio wishes to improve the reproducibility of the work that we publish. This form provides structure for consistency and transparency in reporting. For further information on Nature Portfolio policies, see our Editorial Policies and the Editorial Policy Checklist.

Statistics

For all statistical analyses, confirm that the following items are present in the figure legend, table legend, main text, or Methods section.

n/a	Confirmed	
☐	☒	The exact sample size (*n*) for each experimental group/condition, given as a discrete number and unit of measurement
☐	☒	A statement on whether measurements were taken from distinct samples or whether the same sample was measured repeatedly
☐	☒	The statistical test(s) used AND whether they are one- or two-sided<br>*Only common tests should be described solely by name; describe more complex techniques in the Methods section.*
☐	☒	A description of all covariates tested
☐	☒	A description of any assumptions or corrections, such as tests of normality and adjustment for multiple comparisons
☐	☒	A full description of the statistical parameters including central tendency (e.g. means) or other basic estimates (e.g. regression coefficient) AND variation (e.g. standard deviation) or associated estimates of uncertainty (e.g. confidence intervals)
☒	☐	For null hypothesis testing, the test statistic (e.g. *F*, *t*, *r*) with confidence intervals, effect sizes, degrees of freedom and *P* value noted<br>*Give P values as exact values whenever suitable.*
☒	☐	For Bayesian analysis, information on the choice of priors and Markov chain Monte Carlo settings
☒	☐	For hierarchical and complex designs, identification of the appropriate level for tests and full reporting of outcomes
☒	☐	Estimates of effect sizes (e.g. Cohen's *d*, Pearson's *r*), indicating how they were calculated

Our web collection on statistics for biologists contains articles on many of the points above.

Software and code

Policy information about availability of computer code

Data collection	Custom software for data acquisition and device synchronization. DCAM-API 4.1 for interfacing with camera, PH300 driver to interface with the photon counting module (PicoHarp 300) connected to a SPAD (SPCM-AQ4C, Excelitas).
Data analysis	Custom-built MATLAB code for data processing. The code will be available before publication of the paper.

For manuscripts utilizing custom algorithms or software that are central to the research but not yet described in published literature, software must be made available to editors and reviewers. We strongly encourage code deposition in a community repository (e.g. GitHub). See the Nature Portfolio guidelines for submitting code & software for further information.

Data

Policy information about availability of data

All manuscripts must include a data availability statement. This statement should provide the following information, where applicable:

- Accession codes, unique identifiers, or web links for publicly available datasets
- A description of any restrictions on data availability
- For clinical datasets or third party data, please ensure that the statement adheres to our policy

Data used to generate the main and supplementary figures are available on Figshare (https://doi.org/10.6084/m9.figshare.23811402). All other datasets that support the findings of this study are available from the corresponding author on reasonable request.

Research involving human participants, their data, or biological material

Policy information about studies with human participants or human data. See also policy information about sex, gender (identity/presentation), and sexual orientation and race, ethnicity and racism.

Reporting on sex and gender	Sex and gender were not considered when recruiting participants. Sex and gender was determined based on self report. Consent has been obtained for sharing of individual-level data. For mental subtraction task, seven subjects were recruited which includes 4 male and 3 female participants. One female participant for cuff occlusion study and one male participant for comparing DCS and SCOS were recruited. One male participant was a part of mental subtraction task and comparing DCS and SCOS task. No sex and gender-based analyses were performed.
Reporting on race, ethnicity, or other socially relevant groupings	Race and ethnicity were not considered in the study design for the number of our subjects is small, but we intend to include subjects with all background. Consent has been obtained for sharing of individual-level data.
Population characteristics	See below.
Recruitment	Participants were recruited through on-campus advertisements including flyers.
Ethics oversight	Boston University Institutional Review Board

Note that full information on the approval of the study protocol must also be provided in the manuscript.

Field-specific reporting

Please select the one below that is the best fit for your research. If you are not sure, read the appropriate sections before making your selection.

☐ Life sciences ☒ Behavioural & social sciences ☐ Ecological, evolutionary & environmental sciences

For a reference copy of the document with all sections, see nature.com/documents/nr-reporting-summary-flat.pdf

Behavioural & social sciences study design

All studies must disclose on these points even when the disclosure is negative.

Study description	We quantitatively measure the blood flow change during mental subtraction task using our speckle contrast optical spectroscopy system.
Research sample	Participants were residents in the Boston area.<br>For the mental subtraction task, seven subjects were recruited which includes 4 male and 3 female participants. One female participant for cuff occlusion study and one male participant for comparing DCS and SCOS were recruited. One male participant was a part of mental subtraction task and compared DCS and SCOS tasks.<br>Healthy participants of age group 20-60 with no previous diagnosis or treatment of neurological disorders were recruited.
Sampling strategy	*Describe the sampling procedure (e.g. random, snowball, stratified, convenience). Describe the statistical methods that were used to predetermine sample size OR if no sample-size calculation was performed, describe how sample sizes were chosen and provide a rationale for why these sample sizes are sufficient. For qualitative data, please indicate whether data saturation was considered, and what criteria were used to decide that no further sampling was needed.*
Data collection	*Provide details about the data collection procedure, including the instruments or devices used to record the data (e.g. pen and paper, computer, eye tracker, video or audio equipment) whether anyone was present besides the participant(s) and the researcher, and whether the researcher was blind to experimental condition and/or the study hypothesis during data collection.*
Timing	The study was conducted from July 2022 to Dec 2022.
Data exclusions	Of the seven subjects for mental subtraction task, two did not show lateral frontal lobe activation in fNIRS and thus were excluded from subsequent fiber-based SCOS measurements.
Non-participation	No participants dropped out or declined participation.
Randomization	*If participants were not allocated into experimental groups, state so OR describe how participants were allocated to groups, and if allocation was not random, describe how covariates were controlled.*

Reporting for specific materials, systems and methods

We require information from authors about some types of materials, experimental systems and methods used in many studies. Here, indicate whether each material, system or method listed is relevant to your study. If you are not sure if a list item applies to your research, read the appropriate section before selecting a response.

Materials & experimental systems

n/a	Involved in the study
☒	☐ Antibodies
☒	☐ Eukaryotic cell lines
☒	☐ Palaeontology and archaeology
☒	☐ Animals and other organisms
☒	☐ Clinical data
☒	☐ Dual use research of concern
☒	☐ Plants

Methods

n/a	Involved in the study
☒	☐ ChIP-seq
☒	☐ Flow cytometry
☒	☐ MRI-based neuroimaging
